# Designing a Geodesic Faceted Acoustical Volumetric Array Using a Novel Analytical Method

**DOI:** 10.3390/s23063173

**Published:** 2023-03-16

**Authors:** Taofeek Ayotunde Yusuf, Yongrae Roh

**Affiliations:** School of Mechanical Engineering, Kyungpook National University, Daegu 41566, Republic of Korea

**Keywords:** geodesic-faceted array, spherical array, acoustic transducers, beam patterns, finite element method

## Abstract

We present a novel analytical method as an efficient approach to design a geodesic-faceted array (GFA) for achieving a beam performance equivalent to that of a typical spherical array (SA). GFA is a triangle-based quasi-spherical configuration, which is conventionally created using the icosahedron method imitated from the geodesic dome roof construction process. In this conventional approach, the geodesic triangles have nonuniform geometries due to some distortions that occur during the random icosahedron division process. In this study, we took a paradigm shift from this approach and adopt a new technique to design a GFA that is based on uniform triangles. The characteristic equations that relate the geodesic triangle with a spherical platform were first developed as functions of the operating frequency and geometric parameters of the array. Then, the directional factor was derived to calculate the beam pattern associated with the array. A sample design of GFA for a given underwater sonar imaging system was synthesized through an optimization process. The GFA design was compared with that of a typical SA, and a reduction of 16.5% in the number of array elements was recorded in the GFA at a nearly equivalent performance. Both arrays were modeled, simulated, and analyzed using the finite element method (FEM) to validate the theoretical designs. Comparison of the results showed a high degree of compliance between the FEM and the theoretical method for both arrays. The proposed novel approach is faster and requires fewer computer resources than the FEM. Moreover, this approach is more flexible than the traditional icosahedron method in adjusting geometrical parameters in response to desired performance outputs.

## 1. Introduction

The development of conformal arrays of acoustic transducers has received considerable attention for a long time due to the remarkable advantages of their structures compared with planar arrays. These advantages include a broad beamwidth, wide angular coverage, full integration to the curved surface, space conservation, and reduction in the hydrodynamic dragging effect [1,2,3]. Conformal volumetric arrays on a doubly curved surface similar to a spherical array (SA) are regarded as the most effective geometric configuration for applications requiring a hemispherical beam coverage [4,5]. Consequently, the design of an SA has been a subject of interest to many researchers for decades and numerous studies related to that have been reported in the literature [6,7,8,9,10,11,12,13].

However, despite having excellent performance characteristics, implementation of an SA is difficult because of its complicated and expensive geometry [14,15,16,17]. First, the typically large number of elements, which increases with the radius of the sphere, imposes a considerable economical constraint considering the huge cost of manufacturing a single transducer [18,19]. Second, the uniform distribution of the array elements on the doubly curved surface of the sphere makes the SA geometry complex [20]. Consequently, the geodesic-faceted array (GFA) has become a dominant alternative configuration, employed to achieve a hemispherical coverage equivalent to that of a typical SA [21].

The GFA is a triangle-based quasi-spherical configuration, which is constructed using the icosahedron method imitated from the architectural design technique for geodesic dome roof construction [22,23]. In this method, the geodesic triangles have nonuniform geometries due to the occurrence of some distortions during the random icosahedron division process [24]. Moreover, this approach is a one-off design technique that is inflexible to any systematic modification of the structural parameters. Rather than focusing on the performance requirement, this method is driven by the desired number of triangles. Therefore, the method is unresponsive to any optimization scheme for achieving a specific desired output. Unfortunately, to date, no method is available to improve the design and overcome the glaring disadvantages. Meanwhile, using the conventional computer-aided design approach (i.e., the finite element method (FEM)) to compose a volumetric array geometry such as the GFA typically involves considerable computational complexities, such as a long analysis time and huge computer resources [25]. These factors underscore the need for another remarkably efficient design technique for the GFA.

In this study, we adopted an analytical approach as an efficient alternative to the icosahedron method to synthesize a GFA design using geodesic triangles with uniform geometry throughout the entire volume of the structure. This method enables the rigorous analysis and optimization of the effects of the geometric parameters of the arrays to satisfy the desired performance requirements [26]. Consequently, this method has the prospects of a higher efficiency and flexibility in designing a performance output-driven GFA compared to the icosahedron. Using this novel approach, the geometry of the geodesic triangle was predefined as a function of the operating frequency. The characteristic relationship between the triangles and the sphere was then developed mathematically, followed by the composition of the array. The beam pattern of the GFA was derived as a function of the directional factors to evaluate the performance of the array system based on this geometrical interrelationship. A design case study was conducted through an optimization process using the OptQuest Nonlinear Programming (OQNLP) optimization algorithm considering some given specifications for underwater imaging applications [27]. The performance analysis via this method was compared with that via the FEM to evaluate the efficiency. The novelty of this study lies in the introduction of the analytical method for a more efficient and adaptable configuration of a geodesic acoustical volumetric array compared to the conventional use of the icosahedron.

## 2. Geometry of the Hosting Platform

**Figure 1:**
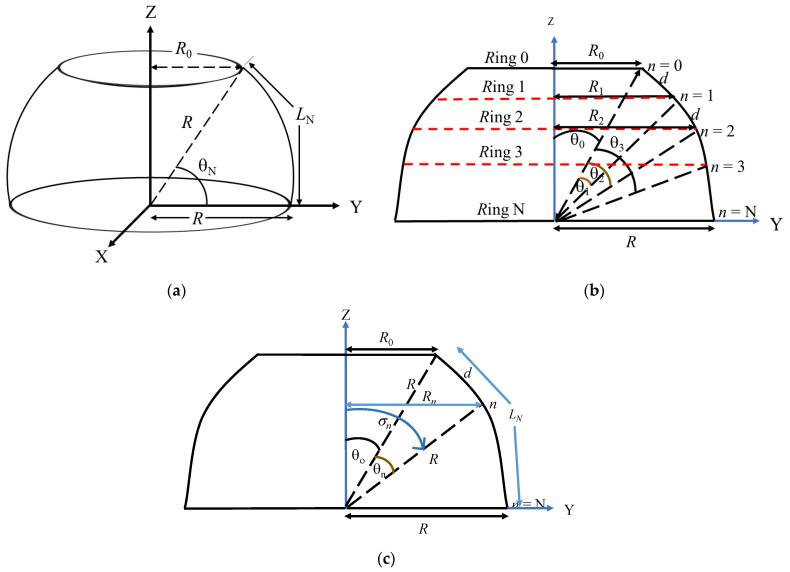
Schematic of the array hosting platform: (**a**) basic; (**b**) simplified analytical; and (**c**) generalized analytical views.

Figure 1a is the schematic representation of the spherical segment as the hosting platform for the GFA. This segment has a radius *R* and a curved surface defined by an arc of length *L_N_* and angle *θ_N_* from its topmost ring of radius *R*_0_ to the great circle. The segment can be extended to a complete sphere by simply modifying the geometrical parameters. Figure 1b shows the analytical view of the platform decomposed into layers of rings of different radii separated by an arc of length *d* and angle *θ*_1_. The ring layers have the order *n* = 0, 1, 2, …., *N*, such as that the total number of rings in the entire array is *N* + 1. The angle *θ*_0_ is the complementary angle to *θ_N_*. Figure 1c presents the generalized version of the geometry showing the *n*th ring of radius *R_n_* at an elevation angle *σ_n_* from the vertical axis Z and arc angle *θ_n_* to the first ring. The mathematical equations representing the relationship between the geometrical parameters are presented in Equations (1) and (2):

(1) $$\left\{\begin{array}{c} \begin{array}{c} \theta_{0} \end{array}\\ R_{0} \end{array}\right\}=\left\{\begin{array}{c} \begin{array}{c} 90-\theta_{N} \end{array}\\ R\sin\theta_{0} \end{array}\right\}\;\mathrm{for}\;n\;=\;0$$

(2) θ1LNθnσnRn=θNNRθNnθ1θ0+θnRsin⁡σn for n > 0

### 2.1. Geometrical Analysis of the Geodesic Faceted Array

**Figure 2:**
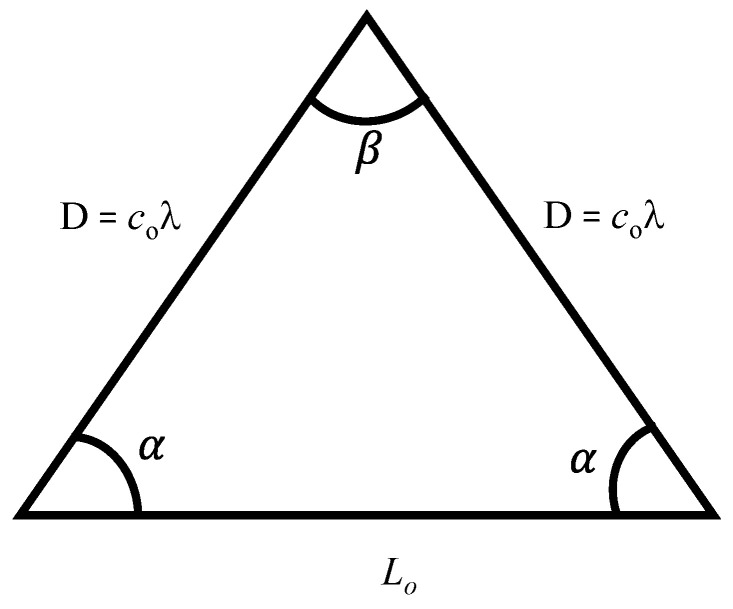
Geometry of the geodesic triangle.

Figure 2 shows the geometry of the proposed geodesic triangle having two equal sides of length *D*, base of length *L_o_*, two equal base angles *α*, and an apex angle *β*. The side length *D* is equal to *c_o_* times *λ*, where *c_o_* is a constant and *λ* is the wavelength of the sound wave, and is related to *L_o_* using Equation (3):

(3) $$L_{o}=2D\cos\alpha$$

**Figure 3:**
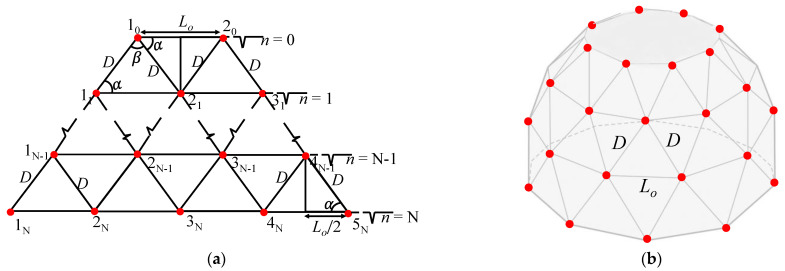

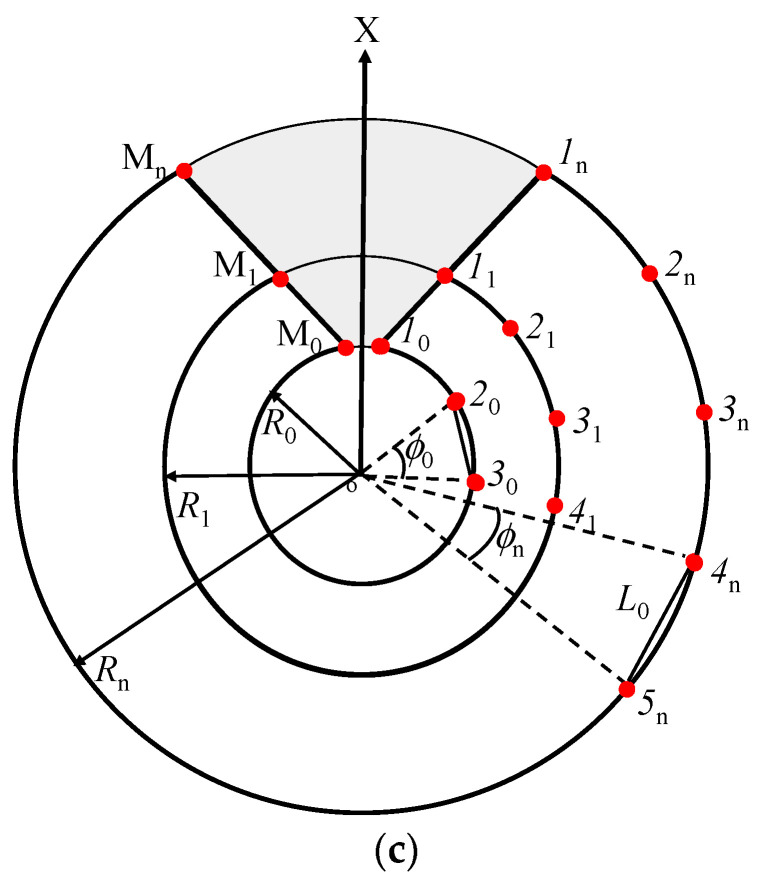
Schematic representation of the GFA: (**a**) rectangular planar; (**b**) typical quasi−spherical; and (**c**) horizontal view.

Figure 3a shows the array of ideal point sources located on the vertices of the geodesic triangles combined and arranged edge-to-edge to establish the entire structure of the GFA. This design facilitates the assemblage of the GFA without constructing the spherical platform. When the arrangement is set to conform to the base circle of the spherical platform, the GFA structurally appears as illustrated in Figure 3b. Figure 3c shows the horizontal view of the distribution of the elements in this configuration. The individual element of the subarray on the *n*th ring evenly separated by an angle *ϕ_n_* is marked by *m_n_*, where *m* represents the order of the elements. Hence, *m_n_* denotes the *m*th element on the *n*th ring while *M_n_* represents the total number of elements on the *n*th ring. The subarrays on the GFA structure are independent of the circumference of each ring but dependent on the geodesic triangles. Consequently, Figure 3b would not complete a full 360° configuration because a certain part of the surface is inevitably left uncovered with elements as depicted by the gray area in Figure 3c. However, this condition is negligible because the small void section can be used as the backside by which the array is attached to the underwater facilities, equipment, or ship as it is in practice [28].

As shown in Figure 3a, the subarray on the topmost ring layer automatically controls the subarrays in the subsequent layers in a regular pattern. Therefore, only the radius of the platform is necessary to determine the number of elements in the topmost layer *M*_0_, as given in Equation (4). The number of elements is an integer; thus, the notation ℵ in Equation (4) denotes the maximum possible integer for the enclosed expression. Consequently, the number of elements in the subsequent layers is determined independently of the radius of each ring by Equation (5), while the total number of elements *N_E_* and the number of triangles *N_T_* required for the implementation of the whole array are given in Equations (6) and (7), respectively.

(4) $$M_{0}=ℵ\left(\frac{2\pi R_{0}}{L_{0}}\right),$$

(5) $$M_{n}=M_{0}+n,$$

(6) $$N_{E}=M_{0}+M_{1}+M_{2}+\ldots {+M}_{N}=\sum_{n=0}^{N}M_{n},$$

(7) $$N_{T}=\left(M_{0}-1\right)+2\sum_{n=1}^{N-1}\left(M_{n}-1\right)+\left(M_{N}-1\right).$$

### 2.2. Characteristic Equations of the Geodesic Faceted Array

**Figure 4:**
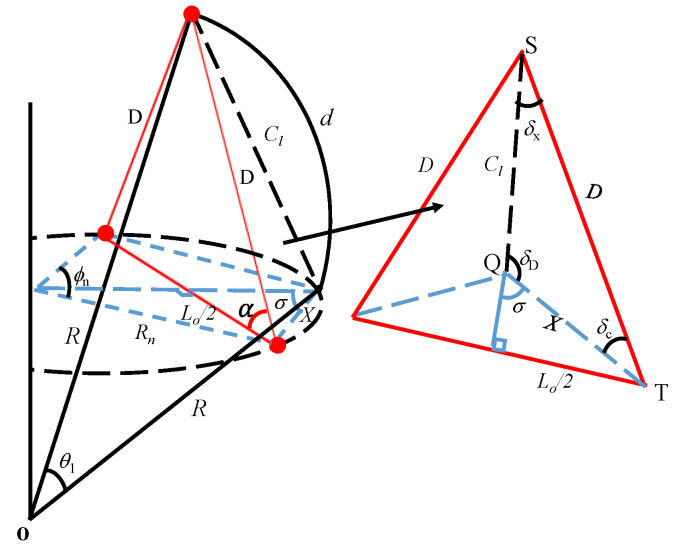
Schematic representation of the geometrical relationship between the geodesic triangle and the platform.

The GFA design concept using the geodesic triangle provides the flexibility to control *L_o_* using only the angle *α*. However, this condition would depend on the vast possibilities to vary *D* within the limit provided by the characteristic equations governing the relationship between the geometry of the triangle and the spherical platform. Figure 4 shows this geometrical connection for deriving the characteristic equations. Figure 4 is a schematic of the geodesic triangle in Figure 2 that is integrated into the doubly curved surfaces of the platform in Figure 1 to synthesize their dependence in the elevation and azimuth directions.

From Figure 4, *R_n_* and *L_o_* can be easily related, as given in Equation (8). The combination of Equation (8) with Equation (3) then yields Equation (9). Applying the trigonometry identities, $\cos\emptyset_{n}=1-2\sin^{2}\left(\frac{\emptyset_{n}}{2}\right)$ and $\cos2\alpha =2\cos^{2}\left(\alpha \right)-1$ yields Equation (10). Furthermore, the chord length on the elevation, $C_{l}=2R\sin\left(\frac{\theta_{1}}{2}\right)$, can be expressed using Equation (2) considering the total arc length of the spherical surface *L_N_* as given in Equation (11). Similarly, the chord length X given in Equation (12) can take the final form as given in Equation (13) when Equation (8) is substituted with the trigonometry identity, $\sin\left(\frac{\emptyset_{n}}{2}\right)=2\sin\left(\frac{\emptyset_{n}}{4}\right)\cos\left(\frac{\emptyset_{n}}{4}\right)$. Using the sine law on the triangle STQ in Figure 4, Equation (14) is obtained subject to the condition that all three interior angles must be real values (i.e., *δ_C_*, *δ_D_*, *δx* ϵ ℜ) according to their respective definitions in Equations (15)–(17). Equations (10) and (14) are the required two characteristic equations for the GFA on the azimuth and elevation planes, respectively, to determine the feasibility of the design.

(8) $$L_{o}=2R_{n}\sin\left(\frac{\emptyset_{n}}{2}\right),$$

(9) $$\frac{D}{R_{n}}=\frac{\sin\left(\frac{\emptyset_{n}}{2}\right)}{\cos\alpha },$$

(10) $$\frac{D}{R_{n}}=\sqrt{\frac{1-\cos\emptyset_{n}}{1+\cos2\alpha }}=\sqrt{\frac{1-\cos\emptyset_{n}}{1-\cos\beta }},$$

(11) $$C_{l}=2R\sin\left(\frac{L_{N}}{2RN}\right),$$

(12) X=Lo2sin⁡σ=Lo2sin⁡90−∅n4,

(13) $$X=\frac{R_{n}\sin\left(\frac{\emptyset_{n}}{2}\right)}{\cos\left(\frac{\emptyset_{n}}{4}\right)}=2R_{n}\sin\left(\frac{\emptyset_{n}}{4}\right),$$

(14) $$D=\frac{\sin\delta_{D}}{\sin\delta_{c}}\cdot C_{l},$$

(15) $$\cos\delta_{C}=\frac{D^{2}+X^{2}-{C_{l}}^{2}}{2DX},$$

(16) $$\cos\delta_{D}=\frac{{C_{l}}^{2}+X^{2}-D^{2}}{2C_{l}X},$$

(17) $$\mathrm{Cos}\delta_{x}=\frac{{C_{l}}^{2}+D^{2}-X^{2}}{2DC_{l}}.$$

### 2.3. Derivation of the Directional Factor of the Array

**Figure 5:**
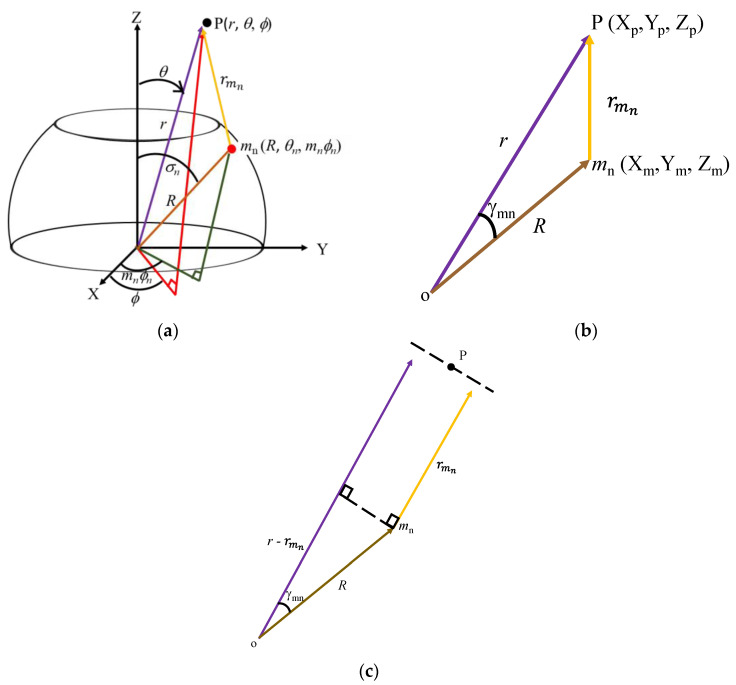
Diagrammatical illustrations for the derivation of the directional factor of an acoustical point source on the spherical segment platform: (**a**) spherical coordinate; (**b**) rectangular coordinate; and (**c**) propagation line parallelism at a far−field distance.

Figure 5a shows a representative element *m_n_* located on the surface of the hosting platform. The acoustic pressure emitted from this element is measured at point P, located at a distance $r_{m_{n}}$ away from the element. This figure shows that the position vectors $\vec{p}$ and $\vec{m_{n}}$ from the origin of point P and *m_n_* have the spherical coordinates (*r*, *θ*, *ϕ*) and (*R*, *θ_n_*, *ϕ_mn_*), respectively. The two position vectors are related as in Equation (18). This equation indicates that the measurement distance $r_{m_{n}}$ is determined as expressed in Equation (19). When the positions of P and *m_n_* are expressed in terms of Cartesian coordinates as shown in Figure 5b, $r_{m_{n}}$ can again be expressed as given in Equation (20). Comparing the two equations, the directional component cos*γ_mn_* would have the value given in Equation (21). Equation (22) is obtained at the far-field where *r* >> *R* and the propagation lines are assumed to be parallel, as illustrated in Figure 5c.

(18) $$\left(\begin{array}{c} \overset{-}{P}\\ \overset{-}{m_{n}} \end{array}\right)=\left(\begin{array}{ccc} X_{p} & Y_{p} & Z_{p}\\ X_{m} & Y_{m} & Z_{m} \end{array}\right)=\left(\begin{array}{ccc} r\sin\theta \cos\emptyset & r\sin\theta \sin\emptyset & r\cos\theta \\ R\sin\sigma_{n}\cos\left({m_{n}\emptyset }_{n}\right) & R\sin\sigma_{n}\sin\left({m_{n}\emptyset }_{n}\right) & R\cos\sigma_{n} \end{array}\right)$$

(19) $$\begin{array}{ll} {r_{m_{n}}}^{2} & ={\left(X_{p}-X_{m}\right)}^{2}+{\left(Y_{p}-Y_{m}\right)}^{2}+{\left(Z_{p}-Z_{m}\right)}^{2}\\ & =r^{2}+R^{2}-2Rr\left\{\sin\theta \sin\sigma_{n}\cos\left(\emptyset -{m_{n}\emptyset }_{n}\right)+\cos\theta \cos\sigma_{n}\right\}. \end{array}$$

(20) $${r_{m_{n}}}^{2}=r^{2}+R^{2}-2Rr\cos\gamma_{mn}$$

(21) $$\cos\gamma_{mn}=\sin\theta \sin\sigma_{n}\cos\left(\emptyset -\emptyset_{mn}\right)+\cos\theta \cos\sigma_{n}$$

(22) $$r_{m_{n}}=r-R\cos\gamma_{mn}$$

The acoustic pressure radiated from this single point source *p* is expressed as given by Equation (23); thus, expressing the pressure from the subarray on the *n*th ring *p_n_* as in Equation (24) is easy [29]. In this equation, *A* is amplitude and *t* is time, while the parameters *k* and *ω* are the wave number and angular frequency, respectively. Consequently, the total acoustic pressure from the array *p_a_* is the summation of the sound pressures *p*_0_, *p*_1_, …, *p_N_* radiated from each subarray on the rings *n* = 0, 1, …, *N* as expressed in Equation (25). Substituting Equation (22) into Equation (25) and applying the far-field condition, that is, 1/$r_{m_{n}}$ ≅ 1/*r* for being *r* >> *R*, Equation (26) is obtained as the final sound pressure from the array.

(23) $$p={\frac{A}{r_{m_{n}}}e}^{j(\omega t-kr_{m_{n}})},$$

(24) $$p_{n}={\frac{A}{r_{1}}e}^{j(\omega t-kr_{1})}+{\frac{A}{r_{2}}e}^{j(\omega t-kr_{2})}+\ldots +{\frac{A}{r_{M_{n}}}e}^{j(\omega t-kr_{M_{n}})}=\sum_{m_{n}=1}^{M_{n}}{\frac{A}{r_{m_{n}}}e}^{j(\omega t-kr_{m_{n}})},$$

(25) $$p_{a}=p_{0}+p_{1}+\ldots +p_{N},\;=\sum_{m_{0}=1}^{M_{0}}{\frac{A}{r_{m_{0}}}e}^{j(\omega t-kr_{m_{0}})}+\sum_{m_{1}=1}^{M_{1}}{\frac{A}{r_{m_{1}}}e}^{j(\omega t-kr_{m_{1}})}+\ldots +\sum_{m_{N}=1}^{M_{N}}{\frac{A}{r_{m_{N}}}e}^{j(\omega t-kr_{m_{N}})}$$

(26) $$p_{a}=\frac{A}{r}e^{j(\omega t-kr)}\sum_{n=0}^{N}\sum_{m_{n}=1}^{M_{n}}e^{jkR\cos\gamma_{mn}}.$$

Setting $\gamma_{mn}=0$, the pressure on the acoustic axis normal to the element *m_n_* is obtained as expressed in Equation (27). $\frac{R}{r}≌0$ at a far-field distance; thus, the maximum pressure amplitude is isolated as given in Equation (28).

(27) $$p_{a}\left(0\right)=\frac{A}{r}e^{j(\omega t-kr)}\sum_{n=0}^{N}\sum_{m_{n}=1}^{M_{n}}e^{jkR}=\frac{A}{r}e^{j\left\{\omega t-kr\left(1-\frac{R}{r}\right)\right\}}\sum_{n=0}^{N}\sum_{m_{n}=1}^{M_{n}}1,$$

(28) $$p_{max}=\frac{A}{r}N_{E}.$$

Dividing the total pressure in Equation (26) by its amplitude in Equation (28) yielded Equation (29). The Heaviside function *H_f_* is incorporated in the expression to remove the backward radiation and its value is determined as given in Equation (30) [30]. The directional factor is the absolute value of the angular-dependent component of this expression as isolated in Equation (31).

(29) $$\frac{p_{a}}{p_{max}}=\frac{1}{N_{E}}e^{j(\omega t-kr)}\sum_{n=0}^{N}\sum_{m_{n}=1}^{M_{n}}e^{jkR\cos\gamma_{mn}}\cdot H_{f},$$

(30) $$H_{f}=\left\{\begin{array}{c} 1,T>0\\ 0,T\leq 0 \end{array}\right.;T=\frac{\left|\cos\gamma_{mn}\right|}{\cos\gamma_{mn}},$$

(31) $$H_{a}\left(\theta ,\emptyset \right)=\left|\frac{1}{N_{E}}\sum_{n=0}^{N}\sum_{m_{n}=1}^{M_{n}}e^{jkR\cos\gamma_{mn}}\cdot H_{f}\right|.$$

**Figure 6:**
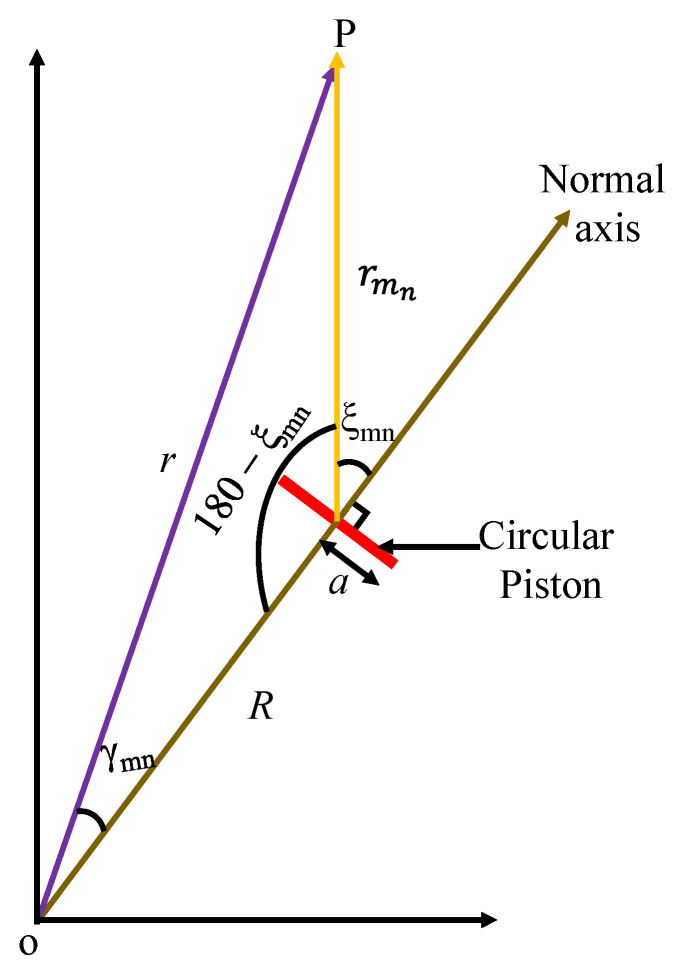
Schematic of the actual circular piston element on the surface of the platform.

As depicted earlier in Figure 2, the point sources are ideally located at the center of the circular piston elements with radiation surfaces of radius *a*. Therefore, the actual acoustic piston element replaces the point source on the platform as shown in Figure 6 to derive its directional factor. The diameter of this element imposes another constraint on the design, that is, *C_l_*, *R_n_ϕ_n_* > 2*a*, due to the inter-element spacing. The angle *ξ_mn_* between the measurement point P and the normal axis of the piston is related with the angle *γ_mn_* at the center of the platform, as given in Equation (32). The relationship shows correspondence in Equation (33) when Equation (22) is substituted and the condition at the far-field distance (i.e., $\frac{R}{r}≌0$) is applied. Consequently, the directional factor of the circular piston element *H_e_* can be expressed as in Equation (34) [29].

(32) $$\begin{array}{l} \frac{r_{m_{n}}}{\sin\gamma_{mn}}=\frac{r}{\sin\left(180-\xi_{mn}\right)}=\frac{r}{\sin\xi_{mn}},\\ \to \frac{r-R\cos\gamma_{mn}}{\sin\gamma_{mn}}=\frac{r}{\sin\xi_{mn}}\\ \to \sin\gamma_{mn}=\sin\xi_{mn}-\frac{R}{r}\cos\gamma_{mn}\sin\xi_{mn} \end{array}$$

(33) $$∴\xi_{mn}=\gamma_{mn},$$

(34) $$H_{e}\left(\theta ,\emptyset \right)=\frac{2J_{1}\left(ka\sin\gamma_{mn}\right)}{ka\sin\gamma_{mn}}.$$

Using the product theorem, the total directional factor of the GFA *H* is calculated as expressed in Equation (35). Using this equation, the beam pattern *b* of the GFA is obtained as shown in Equation (36).

(35) $$H\left(\theta ,\emptyset \right)=\left|H_{a}\left(\theta ,\emptyset \right)\cdot H_{e}\left(\theta ,\emptyset \right)\right|\;=\left|\left[\frac{1}{N_{E}}\sum_{n=0}^{N}\sum_{m_{n}=1}^{M_{n}}e^{jkRcos\gamma_{mn}}\cdot H_{f}\right]\frac{2J_{1}\left(ka\sin\gamma_{mn}\right)}{ka\sin\gamma_{mn}}\right|,$$

(36) $$b\left(\theta ,\emptyset \right)=20\log\left|H\right|.$$

## 3. Design of the Geodesic Faceted Array

Subsequent to the completion of the general design scheme for the development of the novel GFA, the beam pattern in Equation (36) was evaluated with different values of the geometrical parameters *V_i_*, which were critical to the performance of the array where *i* = 1, 2, …, **k**. Here, **k** denotes the total number of such parameters. The values of each of these parameters were varied between the lower and upper bounds *V_i_^L^* and *V_i_^U^*, respectively, to generate additional designs and optimize them for specific beam pattern requirements. These values were combined using the 3**k** method and constituted into predictors, which were fitted against the beam performance outputs *y_j_* to form a nonlinear multiple regression function *y_j_* = *f* (*V_i_^L^*, *V_i_*, *V_i_^U^*), where *j* = 1, 2, …, *h* and *h* is the number of the performance outputs [31]. The optimization process was conducted using the OQNLP algorithm [32].

According to the equations derived in the previous section, the four critical geometrical parameters that influence the beam pattern of the array were identified as the number of layers *N*, radius of elements *a*, the constant ratio of the geodesic triangle’s side length *c_o_*, and the base angle *α*. The efficacy of the beam pattern was illustrated with a sample design of the GFA. The specification for the design case study in terms of the size of the hosting platform and the performance output for the GFA is given in Table 1, considering the requirements for practical underwater imaging systems. The optimization and beam performance evaluation were conducted considering this specific design. All the design calculations and implementation algorithms were written using MATLAB^®^ (version R2019a 9.6) programs.

Based on the performance requirement in Table 1, the objective and constraint conditions were given in Equation (37), while the tolerance of 1° was provided in the upper and lower bounds of the BW of the desired 20°.

Minimize  SLL Subject to:   19° ≤ BW ≤ 21°       SLL ≤ −8 dB                 Elevation ripple level (ERL) ≤ 3 dB                 Azimuth ripple level (ARL) ≤ 3 dB(37)

**Figure 7:**
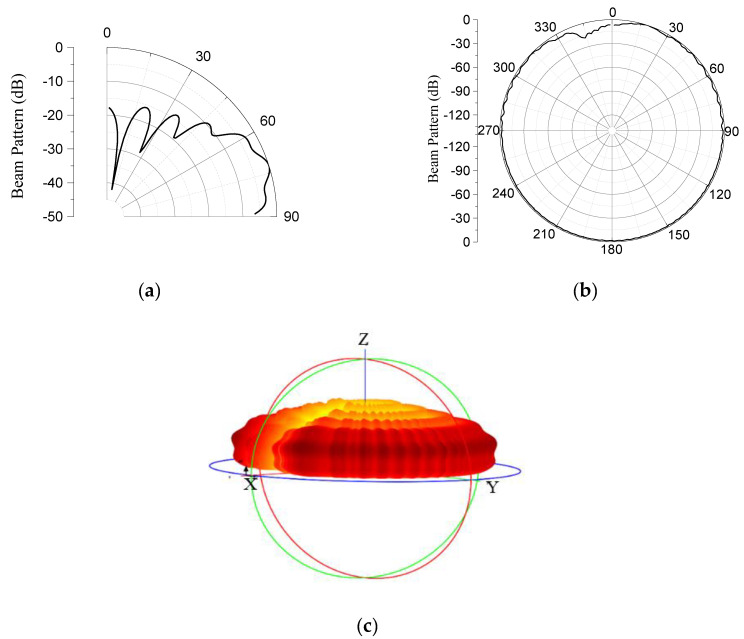
Beam patterns of the GFA on: (**a**) elevation plane; (**b**) azimuth plane; and (**c**) three−dimensional space.

The optimization process was repeatedly conducted for different ranges of values of the geometric parameters. At the penultimate iteration, the final range within which all the target specifications were satisfied was obtained as presented in Table 2. The ‘Basic’ in the middle column of the table represents the penultimate values of the geometrical parameters from which the final lower and upper bounds in the process were found. Table 3 presents the optimized structure, satisfying all the given specifications. The graphical plots of the beam pattern of the model for the elevation plane, azimuth plane, and three-dimensional space are presented in Figure 7a–c, respectively.

## 4. Validation of the GFA Design Using the Finite Element Method

**Figure 8:**
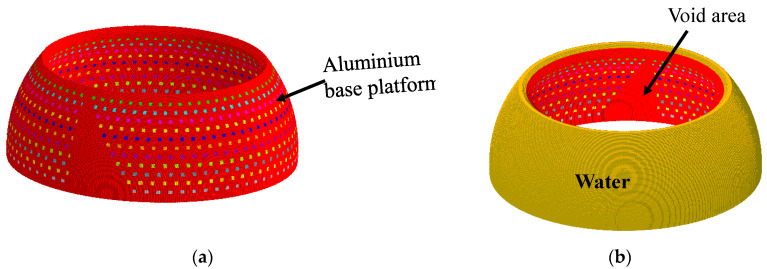
Finite element models of the GFA: (**a**) aluminum base platform; (**b**) array covered with a water layer.

**Figure 9:**
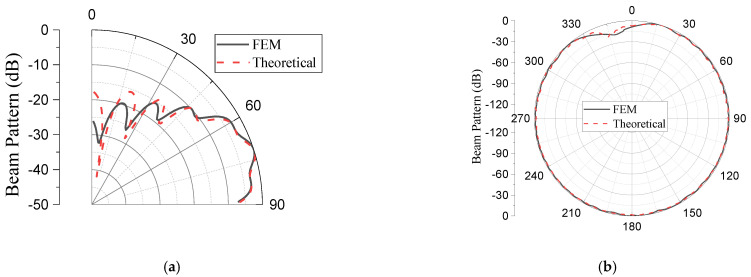
Comparison of the theoretical and finite element analysis beam patterns of the GFA: (**a**) elevation plane; (**b**) azimuth plane.

The validity of the design in Section 3 was verified using the FEM. Using the geometrical parameters obtained theoretically for the sample case, the array was modeled, simulated, and analyzed via the FEM using the commercial software Pzflex^®^ according to the procedures employed by [32]. Figure 8a shows the body of the aluminum base platform incorporating the acoustic piston elements. All the dimensions used were the same as those from the theoretical designs. The outer layer of the model was covered with water to simulate the practical working environments, as shown in Figure 8b. An absorption boundary condition was enforced around the water domain to avoid the reflection of acoustic waves. After discretization of the entire volume, the grid mesh contained 75.4 million elements and 76.0 million nodes. The pressure signals were applied to each of the piston elements for the analysis. The beam patterns from the finite element analysis were compared with the theoretical version, as shown in Figure 9, while the quantitative values of the performance outputs are presented in Table 4. The difference in the peaks of the lower side lobes in Figure 9 was due to the limitation in the size of the elements in the FEM model. However, the difference was inconsequential in the design of the array as long as the main lobe and peak of the highest side lobe coincided. The excellent agreement between the main lobe performances from the two methods can be observed in the table validating the proposed analytical design. Meanwhile, the analytical method in comparison with the FEM demonstrated its merits considering the speed to calculate the performance of the array. Running the model analytically only took 21 min, while the analysis using the FEM took three days and six hours.

## 5. Comparison with a Conventional Spherical Array

**Figure 10:**
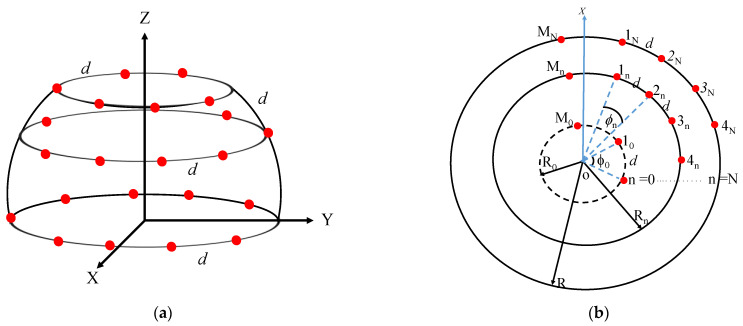
Schematic view of the SA of point sources: (**a**) elevation plane; (**b**) azimuth plane.

Following the design and performance evaluation of the GFA in the previous sections, the design of a typical SA on the same platform was synthesized for the sake of comparison. The geometry of the SA is shown in Figure 10, in which the array elements *m_n_* have a uniform spacing *d* vertically and horizontally on the ring layers. Unlike the GFA, the subarrays strictly depend on the radius of each ring such as that the circumference in each layer is designed for the maximum possible number of elements. This condition implies that the SA is expected to have a higher number of elements than the corresponding GFA design when *L_o_* > *d*. Consequently, the inter-element spacing *d*, the number of elements on each ring *M_n_*, and the total number of elements in the whole array *N_E_* can be defined as given in Equation (38). The number of rings *N* and the radius of the element *a* were selected as the design variables. The optimization and the beam pattern performance analysis were similarly performed.

**Figure 11:**
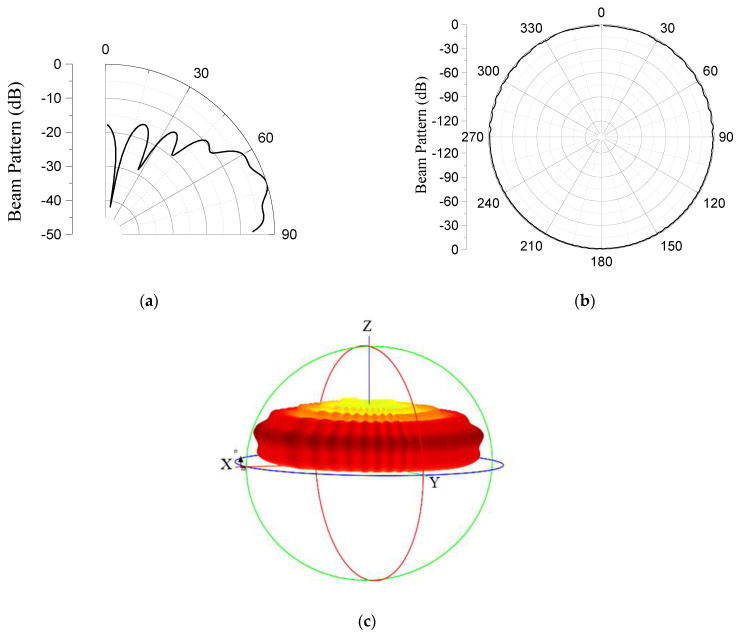
Beam patterns of the SA on: (**a**) elevation plane; (**b**) azimuth plane; and (**c**) three−dimensional space.

The objective function and constraints were set in reference to the performance output of the GFA, as given in Equation (39). The optimization process was also repeated for several iterations until the final range of values of the design variables was obtained as given in Table 5. The final model of the SA that satisfied the desired specification was obtained, as presented in Table 6. Based on Equation (38), the performance on Table 6 was obtained at *d* = 48.9 mm because *R* = 800 mm and *θ_N_* = 35°. The graphical plots of the beam pattern for the elevation plane, azimuth plane, and three-dimensional space are shown in Figure 11a–c, respectively.

(38) d=RθNNMn=ℵ2πRndNE=∑n=0NMn,

Minimize SLL      Subject to: 19° ≤ BW ≤ 21°          SLL ≤ −9.4 dB                     Elevation Ripple level (ERL) ≤ 2.9 dB                     Azimuth Ripple level (ARL) ≤ 2.9 dB(39)

**Figure 12:**
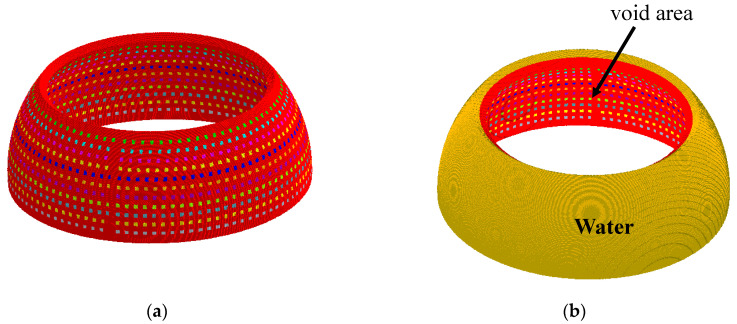
Finite element models of the SA: (**a**) aluminum base platform; and (**b**) array covered with a water layer.

**Figure 13:**
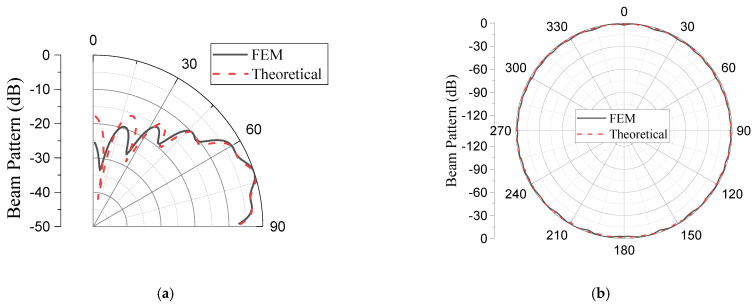
Comparison of the theoretical and finite element analysis beam patterns of the SA: (**a**) elevation plane; (**b**) azimuth plane.

Using the same procedure as in the case of GFA, the SA was modeled using the FEM, as shown in Figure 12. The meshed model contains 76.5 million elements and 77.2 million nodes, and the analysis took approximately three days and five hours. Figure 13 compares the beam patterns from the theoretical analysis with that from the finite element analysis, while the quantitative values of the performance outputs are presented in Table 7.

**Figure 14:**
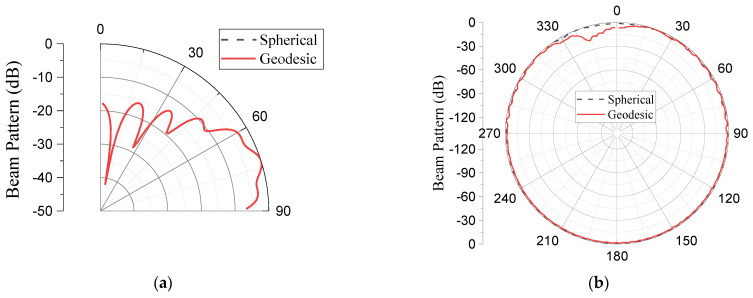
Comparison between the beam patterns of the SA and GFA: (**a**) elevation plane; (**b**) azimuth plane.

Table 8 shows a quantitative comparison between the SA and the GFA, while Figure 14 compares the beam patterns. The small gap at the terminal parts of the azimuth beam in Figure 14b is due to the void section at the backside of the spherical segment for the GFA design. With 880 elements against 1054, the GFA significantly reduced the number of elements in the SA by 16.5%, while the performance is virtually the same, as shown in the table. This reduced number of elements also explains the slightly low azimuth ripple level in the SA. The reduced ripple level is associated with a narrow element spacing or a dense element grid [1].

Consequently, the proposed analytical method improves the efficiency of the GFA design by developing triangles with uniform geometry. This is a novelty compared with the random shapes and sizes of the geodesic triangles in the conventional icosahedron method. The method also enhances structural flexibility, increasing the adaptability of the design to the desired or output-driven performance compared with the one-off approach of the icosahedron method. Finally, the analysis of the GFA via the proposed method is approximately 210 times faster than the FEM.

## 6. Conclusions

GFA is an excellent alternative volumetric array structure to circumvent the challenges associated with an SA without compromising its excellent performance. However, an exact method to design a GFA is currently unavailable. Thus, this study was conceived to improve the design technique of GFA by using an analytical method, which can act as a substitute for the existing traditional icosahedron approach. Unlike the latter, the novel GFA developed in this work via the new analytical method is based on triangles with uniform, defined, and predetermined geometries throughout the entire volume of the array. The new method provides the flexibility to adjust geometrical parameters in response to desired performance outputs compared to the one-off approach of the icosahedron method. Moreover, analysis via the proposed method is faster than computer-aided techniques such as the FEM. The proposed method also preserves the intrinsic property of GFA by reducing the acoustical elements, which could have been used in a typical SA. Consequently, the method is proven to be more efficient, flexible, and time saving than the icosahedron method.

## Figures and Tables

**Table 1:** Specifications for a design case study.

	Parameter	Specification
Size of the platform	Radius, *R*	800 mm
	Arc angle, *θ_N_*	35°
Performance requirements	Elevation half-power (−3 dB) Beamwidth (BW)	20° [33]
	Elevation side lobe level (SLL)	≤−8 dB [34]
	Ripple level	≤3 dB
	Center frequency	16 kHz [35]

**Table 2:** Final range of geometrical parameters for the GFA.

Geometrical Parameter	Lower Bound	Basic	Upper Bound
*N*	7	9	11
*a* (mm)	9	12	15
α (^o^)	59	60	61
*c_o_*	0.58	0.60	0.62

**Table 3:** Structure of the optimized GFA model.

**Geometrical parameter**	*N*	10
	*a* (mm)	10.0
	*α* (^o^)	61.0
	*c_o_*	0.6
**Performance output**	BW (^o^)	20.6
	SLL (dB)	−9.4
	ERL (dB)	2.9
	ARL (dB)	2.9

**Table 4:** Comparative values of the performance outputs of the theoretical and finite element analysis beam patterns of the GFA.

Methods	Performance Outputs			
	BW (°)	SLL (dB)	ERL (dB)	ARL (dB)
Theoretical	20.6	−9.4	2.9	2.9
FEM	20.5	−9.9	2.9	3.0

**Table 5:** Final range of values for the design variables for SA.

Design Variable	Lower Bound	Basic	Upper Bound
*N*	7	9	11
*a* (mm)	9	12	15

**Table 6:** Final structure of the SA model.

**Design variable**	*N*	10
	*a* (mm)	10.0
**Performance output**	BW (°)	20.6
	SLL (dB)	−9.4
	ERL (dB)	2.9
	ARL (dB)	2.8

**Table 7:** Quantitative values of the performance outputs from the theoretical and finite element analysis beam patterns of the SA.

Methods	Performance Outputs			
	BW (°)	SLL (dB)	ERL (dB)	ARL (dB)
Theoretical	20.6	−9.4	2.9	2.8
FEM	20.6	−9.9	2.9	2.9

**Table 8:** Quantitative comparison between the SA and GFA.

Model	Structural Parameters				Performance Parameters					
	*R* (mm)	*θ_N_* (^o^)	*N*	*a* (mm)	BW (^o^)	SLL (dB)	ERL (dB)	ARL (dB)	*N_E_*	% Element Reduction
Spherical	800	35	10	10	20.6	−9.4	2.9	2.8	1054	--
Geodesic	800	35	10	10	20.6	−9.4	2.9	2.9	880	16.5

## Data Availability

Not applicable.

## References

[1] Josefsson L., Persson P., El-Hawary M.E. (2006). Conformal array antenna theory and design. IEEE Press Series on Electromagnetic Wave Theory.

[2] Munno F. (2021). Conformal array geometry for hemispherical coverage. Electronics.

[3] Park J., Lim H.J., Trinh-Van S., Park D., Jung Y.K., Lim D., Hwang K.C. (2022). Derivation of a universally valid array factor of a conformal arrays based on phase compensation and genetic learning particle swarm optimization. Appl. Sci..

[4] Baee R.K., Forooraghi K., Chamaani S. (2012). Conformal array pattern synthesis using a hybrid WARP/2LB-MOPSO algorithm. Int. J. Antennas Propag..

[5] Tomasic B., Turtle J., Liu S. Spherical arrays-design considerations. Proceedings of the 2005 18th International Conference on Applied Electromagnetics and Communications.

[6] Wong K.T., Morris Z.N., Nnonyelu C.J. (2019). Rules-of-thumb to design a uniform spherical array for direction finding—Its Cramér–Rao bounds’ nonlinear dependence on the number of sensors. J. Acoust. Soc. Am..

[7] Yang Y., Chu Z., Shen L., Xu Z. (2016). Functional delay and sum beamforming for three-dimensional acoustic source identification with solid spherical arrays. J. Sound Vib..

[8] Huang D. (2017). Theory and numerical simulation for a design of broadband constant beam pattern transducer. J. Acoust. Soc. Am..

[9] Zhang Y., Wang L., Qin L., Zhong C., Hao S. (2021). Spherical-omnidirectional piezoelectric composite transducer for high- frequency underwater acoustics. IEEE Trans. Ultrason. Ferroelectr. Freq. Control..

[10] Nagaoka R., Tabata T., Takagi R., Yoshizawa S., Umemura S.I., Saijo Y. (2017). Development of real-time 3-D photoacoustic imaging system employing spherically curved array transducer. IEEE Trans. Ultrason. Ferroelectr. Freq. Control..

[11] Yang B., Shi S., Yang D. (2019). Acoustic source localization using the open spherical microphone array in the low-frequency range. MATEC Web Conf..

[12] Radivojević V.M., Rupčić S., Grgić K. (2017). Radiation pattern optimisation of an antenna array on the spherical surface by using a varying number of optimisation parameters. IET Microw. Antennas Propag..

[13] Hayashi E., Kanno N., Shintate R., Ishii T., Nagaoka R., Saijo Y. (2022). 3D ultrasound imaging by synthetic transmit aperture beamforming using a spherically curved array transducer. Jpn. J. Appl. Phys..

[14] Sadeghpour S., Meyers S., Kruth J.P., Vleugels J., Kraft M., Puers R. (2019). Resonating shell: A spherical-omnidirectional ultrasound transducer for underwater sensor networks. Sensors.

[15] Saqib N.U., Khan I.A. (2015). hybrid antenna array design for 3-D direction of arrival estimation. PLoS ONE.

[16] Huong N.T., Sabban A. (2020). Beamforming phased array antenna toward indoor positioning applications. Advance Radio Frequency Antennas for Modern Communication and Medical Systems.

[17] Tomasic B., Turtle J., Liu S., Schmier R., Bharj S., Oleski P. The geodesic dome phased array antenna for satellite control and communication-subarray design, development and demonstration. Proceedings of the IEEE International Symposium on Phased Array Systems and Technology.

[18] Goossens R., Bogaert I., Rogier H. (2009). Phase-mode processing for spherical antenna arrays with a finite number of antenna elements and including mutual coupling. IEEE Trans. Antennas Propag..

[19] Daher E.B. (2016). Analysis and Design of Nonuniform Arrays for Direction Finding. Ph.D. Thesis.

[20] Du C., Leclere Q., Li B. Design and Evaluation of Open Spherical Microphone Arrays. Proceedings of the 24th International Congress on Sound Vibration.

[21] Dianfei P., Yanshan B., Naiping C. (2015). Hemispherical Coverage Array Antenna and Performance Analysis. Wirel. Pers. Commun..

[22] Zhang J., Liu P., Zhuang Z., Wei J., Liu X., Wang H., Li L. Design of a Spherical Conformal Phased Array Antenna Based on the Truncated Icosahedron. Proceedings of the 2020 IEEE Radar Conference.

[23] Grech C., Azzopardi M.A., Buttigieg V. Circular Lattice Design for UHF Geodesic Dome Phased Array Antenna with Reduced Footprint. Proceedings of the 2020 International Symposium on Antennas and Propagation.

[24] Qiu Z., Habeshaw R., Fortine J., Huang Z., Démoré C., Cochran S. (2012). New piezocrystal material in the development of a 96-element array transducer for MR-guided focused ultrasound surgery. AIP Conf. Proc..

[25] Sim M.J., Hong C., Jeong W.B. (2021). Hybrid equivalent circuit/finite element/boundary element modeling for effective analysis of an acoustic transducer array with flexible surrounding structures. Appl. Sci..

[26] Pelham T.G., Hilton G., Mellios E., Lewis R. (2021). Conformal antenna array design using aperture synthesis and on-platform modeling. IEEE Access.

[27] Lasdon L.S., Plummer J.C. (2008). Multistart algorithms for seeking feasibility. Comput. Oper. Res..

[28] De Witte E. (2007). Design and Development of Spherical Array Antennas. Ph.D. Thesis.

[29] Kinsler L.E., Frey A.R., Coppens A.B., Sanders J.V. (2000). Fundamentals of Acoustics.

[30] Kim H., Roh Y. (2010). Analysis of the radiation pattern of conformal array transducers. J. Acoust. Soc. Korea.

[31] Koukouvinos C. (1996). Orthogonal 2k and 3k factorial designs constructed using sequences with zero autocorrelation. Stat. Probab. Lett..

[32] Yusuf T.A., Pyo S., Roh Y. (2021). A novel versatile approach for underwater conformal volumetric array design. Sensors.

[33] Gerlotto F., Georgakarakos S., Eriksen P.K. (2000). The application of multibeam sonar technology for quantitative estimates of fish density in shallow water acoustic surveys. Aquat. Living Resour..

[34] Baron V., Finez A., Bouley S., Fayet F., Mars J.I., Nicolas B. (2021). Hydrophone array optimization, conception, and validation for localization of acoustic sources in deep-sea mining. IEEE J. Ocean. Eng..

[35] Nishimura C.E. (1997). Fundamentals of Acoustic Backscatter Imagery.

